# Costs and benefits of interventions aimed at major infectious disease threats: lessons from the literature

**DOI:** 10.1007/s10198-020-01218-4

**Published:** 2020-08-13

**Authors:** Klas Kellerborg, Werner Brouwer, Pieter van Baal

**Affiliations:** Erasmus School of Health Policy and Management, Erasmus University Rotterdam, Rotterdam, The Netherlands

**Keywords:** Literature review, Health economics, Economic evaluations, Infectious diseases, Future costs, I190, I180

## Abstract

Pandemics and major outbreaks have the potential to cause large health losses and major economic costs. To prioritize between preventive and responsive interventions, it is important to understand the costs and health losses interventions may prevent. We review the literature, investigating the type of studies performed, the costs and benefits included, and the methods employed against perceived major outbreak threats. We searched PubMed and SCOPUS for studies concerning the outbreaks of SARS in 2003, H5N1 in 2003, H1N1 in 2009, Cholera in Haiti in 2010, MERS-CoV in 2013, H7N9 in 2013, and Ebola in West-Africa in 2014. We screened titles and abstracts of papers, and subsequently examined remaining full-text papers. Data were extracted according to a pre-constructed protocol. We included 34 studies of which the majority evaluated interventions related to the H1N1 outbreak in a high-income setting. Most interventions concerned pharmaceuticals. Included costs and benefits, as well as the methods applied, varied substantially between studies. Most studies used a short time horizon and did not include future costs and benefits. We found substantial variation in the included elements and methods used. Policymakers need to be aware of this and the bias toward high-income countries and pharmaceutical interventions, which hampers generalizability. More standardization of included elements, methodology, and reporting would improve economic evaluations and their usefulness for policy.

**Electronic supplementary material:**

The online version of this article (10.1007/s10198-020-01218-4) contains supplementary material, which is available to authorized users.

## Introduction

Historically, infectious disease outbreaks have proven to be potentially devastating. A prominent example is the Spanish influenza which may have claimed as many as 50 million lives [1]. The number of outbreaks of infectious diseases has been increasing since 1980, as has the number of unique pathogens [2]. To prevent and effectively combat outbreaks, reporting agreements such as those arranged in the International Health Regulations (IHR) between national governments and international organizations were established [3]. The current IHR require the countries which ratified them to develop a minimum capacity of core functions related to surveillance and response [3]. However, with new threats emerging and given the fragile health systems in many parts of the world, outbreaks still have the potential to occur with potentially severe consequences in multiple countries. Therefore, there is a continuous pressure to improve available detection and response systems, and to increase the possibilities of preventing new threats from doing too much harm.

A recent example that illustrates the relevance of outbreak containment is the Ebola outbreak of 2014. The response to this outbreak received important criticisms, and as a consequence, the World Health Organization reformed, improving its response to infectious threats [4]. Aside from international organizations and non-governmental organizations, under the IHR nations are obliged to have at least a minimum threat handling capacity. However, countries are usually faced with limited healthcare budgets, which require prioritization of what to fund and in which disease areas to invest. Funding of detection and response facilities in case of an outbreak also needs to compete for available resources. Preferably, decisions on how to optimally allocate scarce healthcare resources are informed by sound estimates of potential costs and benefits of various policy scenarios. Assessing the cost-effectiveness of different prevention and treatment strategies is of utmost importance to ensure value for money and optimal health and welfare from the available budgets [5]. However, obtaining sound estimates of both costs and effects of intervention strategies, compared to a relevant comparator (such as the current situation or doing nothing), is not a straightforward task, and one that is full of methodological challenges.

To comprehensively capture the costs and benefits related to an intervention, numerous issues need to be considered, including the costs of the intervention itself, the incurred and avoided health losses, and the incurred and avoided treatment costs. A full analysis may also include elements such as production losses due to illness and premature death from the disease, or even broader economic impacts such as those due to reduced trade and tourism. Clearly, some of these elements may be more difficult to estimate and quantify. Importantly, in applied cost-effectiveness analyses, the decision regarding which costs to include depends on the perspective chosen. The societal perspective aims to capture all relevant costs and effects, regardless of where, when or on whom in society they fall [6]. Narrower perspectives, such as the patient’s perspective or a healthcare perspective, are sometimes used, which limits the scope of the evaluation. Especially for interventions targeted at preventing outbreaks, which can have rather broad impacts, adopting a societal perspective seems warranted [7]. Indeed, the impact of outbreaks is not confined to the healthcare sector and interventions to prevent or mitigate these outbreaks are often not confined to healthcare interventions (or funding). Note that when evaluating pandemics not only a broad range of cost categories in various sectors of the economy need to be considered but also the fact that a pandemic may trigger non-marginal changes in the healthcare sector and possibly the entire economy. Non-marginal changes in the health sector may occur when outbreaks cause capacity problems and displace a large portion of usual care within healthcare and outside the healthcare sector entire industries might be threatened. This suggests that the usual micro-economic perspective which is taken in economic evaluations is insufficient and a more macro-economic perspective might be more [8, 9].

Simulation models are often used to estimate the consequences of preventing or mitigating disease outbreaks [10]. Modeling of infectious diseases is typically done using either so-called static or dynamic transmission models [11]. Static models, such as decision trees and Markov models, assume that the probability of infection between individuals is constant over time. Dynamic models allow for the force of infection to be varied, and can include possible herd immunity effects [12]. Dynamic models are often considered to be more complex, but may be preferred to static models because they are able to take into account a varying transmission rate, which is highly relevant in this context [11]. Both types of models offer the ability to model different scenarios and interventions, and costs and benefits can be estimated using these models by linking them to events and/or states distinguished in the model [11].

An important challenge in infectious disease modeling is to account for behavioral responses that occur when under the threat of an infection [13, 14]. Whether or not individuals themselves take action in the face of an outbreak (threat) may introduce bias in the evaluation of a policy to mitigate an outbreak [15]. For instance, when the actual severity and the perceived severity of an illness diverge, this may complicate forecasts of the impact of interventions. Apart from the challenges in modeling the disease itself, there is also room for improvement in other parts of infectious outbreak policy evaluation. Previous research indicated that outbreak evaluations are often biased toward high-income settings and that little research is done in low-income regions [14]. High-income and low-income countries may face a different set of challenges, including different resource and capacity constraints, different threats and different living environments. Such differences need to be accounted for in evaluations and when attempting to translate results of interventions across settings. Furthermore, it should be acknowledged that an intervention, like setting up a surveillance system or response protocol, targeted at one specific disease may strengthen the healthcare system more generally. This means that the effects of such a measure could go beyond preventing and mitigating one particular type of outbreak. Such “policy spill-over effects” are rarely included [16].

The aim of this study is to review cost-effectiveness studies of major outbreak threats, based on WHO publications [17]. The focus of this review will be on investigating the methodological approaches used to estimate costs and (health) benefits, with the aim of improving our understanding of how evaluations of interventions related to outbreaks are currently conducted. This is key, because if decisions are to be based on available evidence, the evidence itself should preferably be comparable, valid and broad enough for policymakers to consider all relevant elements in the decision-making process.

## Methods

To determine how costs and benefits in economic evaluations of interventions aimed at (potential) outbreaks are estimated, we first compiled a list of major outbreak threats of the 21st century. We based this on publications of the WHO which were produced for the meeting ‘’Anticipating Emerging Infectious Disease Epidemics’ [17]. The aim of selecting diseases based on this list was not to capture the most severe diseases or those that, in retrospect, turned out to be found the most costly outbreaks, rather we aimed to collect a broad sample of diseases that have the potential of causing large-scale health and economic damage. Future major outbreaks may have similar characteristics to their predecessors, implying that policy decisions regarding preventing or countering them will (need to) be based on similar information as found in the economic evaluations included here. In this review, we extracted information on study outcomes and methods, using a pre-determined protocol.

### Data

We searched PubMed and SCOPUS in April 2018 for the following major outbreaks in the 21st century; SARS in 2003, H5N1 in 2003, H1N1 in 2009, Cholera in Haiti in 2010, MERS-CoV in 2013, H7N9 in 2013 and the West African Ebola outbreak in 2014. For this search, we constructed three blocks, which we used in combination and all terms were searched for in title and/or abstract. The full syntax for both Pubmed and SCOPUS is available in Appendix 1. The first block was the list of the relevant diseases in various combinations: Middle East respiratory syndrome coronavirus OR SARS OR H5N1OR H1N1 OR Cholera OR MERS-CoV OR H7N9 OR Ebola. The second block defined the study type: economic OR cost* OR costing. The third block complemented the second: benefits OR effectiveness OR cost-effectiveness OR cost–benefit OR cost-utility. Last, filters were applied to include studies from 2003 and onward and exclude studies with only animal subjects. We only considered articles published from 2003, given that we focused on the outbreaks of 2003 and later. We assumed that no articles had been published on the relevant outbreaks before their occurrence.

### Study selection

We performed two screening rounds. In the first round, we screened articles based on title and abstract. In the second round, we screened full-text articles. Studies reviewed in full-text, but subsequently excluded, are shown with a justification for their exclusion in Appendix 2. We included peer-reviewed studies that conducted a quantitative economic evaluation of any form (cost-minimization, cost-effectiveness, cost-utility, or cost–benefit evaluations) with one or more comparators, and evaluated one or more interventions within the context of the outbreaks previously mentioned. We not only included studies based on actual reported case data but also included studies using measures of how infectious a disease is based on observations to model the outbreak, for example force of infection. We excluded review papers and only included studies written in English.

### Data extraction and analysis

The in-depth reviewing of the selected studies focused on characteristics of the study setting (target disease, country, interventions evaluated), issues related to modeling, and finally, the included costs and health gains. We will elaborate on the latter two.

We extracted information about what type of model (dynamic or static) was used in the included studies, and how the studies dealt with uncertainty around estimates. Some models, such as microsimulations, are stochastic by definition while other models may employ various types of sensitivity analyses. Sensitivity analyses may be used not only to test uncertainties, but also to test different assumptions of the transmission model and the economic model. Such analyses may involve varying assumptions and parameters related to the specific setting of a study, which can inform the generalizability of the results to other settings, for instance other drug prices or intervention efficacies [18]. Thus, we also extracted information about the setting of the included studies and grouped these settings according to the World Bank Country and Lending Groups [19].

We divided costs into two categories: (1) costs that occur within the healthcare sector and (2) costs that occur outside of the healthcare sector. For both categories, we further divided the costs into short-term costs and future costs. We defined short-term cost as the costs that occur during the outbreak, and the future costs as those that occur when life is extended. Short-term costs within the healthcare sector are for example staff, equipment, and current treatment costs. Future costs within the healthcare sector include both future consumption of healthcare related to the specific disease being targeted and also future utilization of healthcare due to other diseases in life years gained [20].

Short-term costs outside the healthcare sector are costs that arise for example for the patient or the caregiver of a patient. These costs can be for transportation, time off from work to undergo treatment in a healthcare facility, or out-of-pocket expenses. Future costs outside the healthcare sector include productivity losses due to disability and premature mortality. Productivity losses are often estimated by methods such as the Human capital approach or the Friction cost method. The human capital approach quantifies the remaining productivity that would have occurred during all life years lost [21]. The friction cost method quantifies the time required to replace a worker by someone else, like a formerly unemployed person [22].

There is currently an ongoing debate on which future costs to include in health economic evaluations [23]. This particularly relates to costs in gained life years (i.e., those years that patients would not have lived without the intervention, but do with). If the aim is to comprehensively capture all impacts of an intervention, future costs and benefits, related to consumption and production, cannot be excluded from an analysis [20, 24].

For all cost categories distinguished we extracted information regarding the measurement and valuation of these costs and categorized them according to a micro-costing or a gross-costing approach. Micro-costing refers to the approach of costs’ estimation where the unit cost is multiplied by the used quantity of the referred unit, gross-costing; on the other hand, is when a budget is divided into sectors of usage [25]. Micro-costing is considered a more precise estimation of cost but may be more demanding in terms of data availability, and the sum may even exceed the total budget [25]. Gross costing is less data demanding but may misclassify costs between sectors. Finally, we checked whether studies took account of more disruptive effects on the healthcare sector and the wider economics to account for non-marginal impacts of a pandemic.

To fully account for all the relevant effects, the time horizon should be long enough to capture all costs and benefits of the intervention. Therefore, we extracted this information from the included articles. In addition, we extracted information about discounting of cost and health effects. Discounting is common in economic evaluations as the effects that occur in the present are valued higher than similar effects occurring in the future. The WHO-CHOICE uses an annual discount rate of 3% for both health effects and costs, but national guidelines may recommend different rate(s) [26].

## Results

**Fig. 1:**
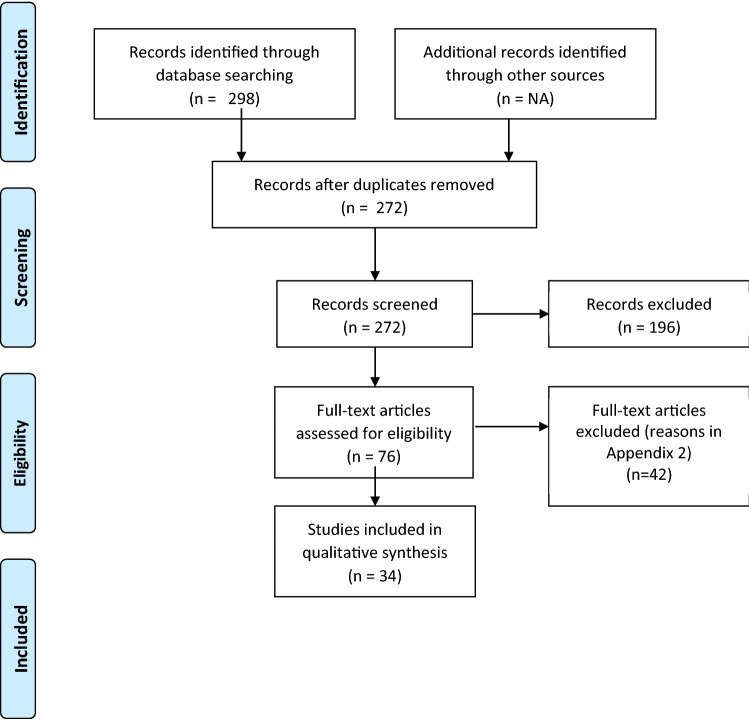
Schematic flowchart of study selection process

The literature search resulted in 298 records, of which 76 met the inclusion criteria and were assessed in full-text. Of the 76 records, 34 were considered eligible for inclusion in our study. The 42 excluded records were excluded due to not conducting any form of economic evaluation (10 records), methodology paper (6 records), not based on relevant outbreaks (4 records), effectiveness study (3 records), not in English (3 records), studying animal subjects (3 records), not quantifying the impact of an intervention against outbreak (3 records), reviews (2 records), not comparing intervention against baseline (1 record), being a preliminary study to an already included study (1 record), budget impact analysis (1 record), and not able to access (5 records) (Fig. 1).

As shown in Table 1, H1N1 was the most frequently studied outbreak, with 29 of the included studies. Few studies compared more than two interventions. Pharmaceutical interventions (vaccinations and antivirals) were studied in 23 included studies. Vaccinations were most commonly studied, followed by school closure. Evaluated non-pharmaceutical interventions mostly consisted of strategies aimed at decreasing contact between infected and susceptible individuals. Only four studies compared pharmaceutical interventions with non-pharmaceutical interventions.

**Table 1:** Sample descriptive

Outbreak	Frequency^a^	%^a^
H1N1	29	85
H5N1	3	9
SARS	3	9
Ebola	1	3
H7N9	1	3

Intervention	Frequency^a^	%^a^
Vaccination	16	47
School closure	8	24
Antivirals	6	18
Quarantine	2	6
Personal Protective Equipment	2	6
Social distancing	2	6
Screening	1	3
Whole response program	1	3
Sick leave policies	1	3
Non-specified non-pharmaceutical	1	3
Other pharmaceutical	1	3

Setting^b^	Frequency	%
High income	29	85
Upper-middle income	4	12
Low income	1	3

^a^Sum of frequencies and/or percentages larger than number of studies included as some studies evaluated more than one outbreak/intervention

^b^ Classified accordingly to the World Bank’s classification of Countries and Lending Groups [19]

Of the included studies, 17 were cost-effectiveness analyses [27–42]. Cost-utility analyses were performed in 13 studies [43–55], and four studies performed cost–benefit analyses [56–59]. 29 studies were conducted in a high-income setting, 4 were conducted in an ‘upper-middle’ income setting and only one was conducted in a low-income setting. Of the high-income studies, a majority (i.e., 16 out of 29) were situated in the US (Table 2).

**Table 2:** Overview of included articles

Author	Type	Setting	Outbreak	Intervention	Results summary	Model type	Uncertainty	Perspective stated	Time horizon stated	Costs					Health outcome	Discount rate (%)
										Within HC		Outside HC		Costing method		
										Short term	Future	Short term	Future			
Basurto-Davila [56]	CBA	US	H1N1	Vaccination	Vaccination averted 4600 influenza cases and was cost-saving	Dynamic	Probabilistic	Societal	NR	T,ADM,EQ	Not included	AB	FNM	Micro-costing	Cases averted	3
Brown [57]	CBA	US	H1N1	School closure	Cost per averted case with a 8-week school closure varied between 14,000 and 25,000 depending on the infection rate	Dynamic	Univariate	Societal	NR	T	Not included	AB	FNM	Mixed	Cases averted	3
Mamma [58]	CBA	Greece	H1N1	Vaccination	Depending on participation rate, % symptomatic the net cost per case averted ranged from -36.67 to 35.42 EUROs	Static	Univariate	NR	NR	T	Not included	AB	Not included	Micro-costing	Cases averted	NR
Wang [59]	CBA	China	H1N1	Combination of preventive measures, testing and treatment based on polices enacted in Hubei Province	The estimated benefits of the Hubei response program were more than five times the estimated costs.	Static/mathematical	–	NR	NR	T,ADM	Not included	AB	FNM	Micro-costing	Cases averted	NR
Tracht [60]	CEA^a^	US	H1N1	PPE	10%, 25% and 50% use of facemasks in the population could reduce costs by 478, 570, 573 billion USD respectively and decrease the number of cases	Dynamic	Univariate	NR	NR	T,ADM	Not included	AB	FNM	Micro-costing	Cases averted	NR
Lee2 [27]	CEA^a^	US	H1N1	Vaccination	The cost per case averted varied between 14 and 2387 USD f depending on vaccine cost and vaccination time.	Static	Probabilistic	Patient	NR	T	Not included	AB	Not included	Micro-costing	Cases averted	3
Andradóttir [28]	CEA^a^	US	H1N1	vaccination, antiviral, school closure, social distancing	Many scenarios consisting of combinations of interventions are presented. Most scenarios resulted in lower attack rates and cost-savings.	Dynamic	Univariate	NR	NR	T, CP	Not included	AB	FNM	Micro-costing	Attack rates	NR
Brouwers [35]	CEA	Sweden	H1N1	Vaccination	A vaccination rate of 60% of the population was the most cost-effective saving 2.5 billion SEK	Dynamic	Univariate	Societal	NR	T,ADM	Not included	AB	Not included	Mixed	Cases averted	NR
Carias [36]	CEA	west Africa	Ebola	Other pharmaceutical	Administration of malaria treatment to Ebola admitted patients dominated no malaria treatment resulting in fewer cases and cost-savings	Dynamic	Probabilistic	Healthcare	1-year	T,ADM,EQ	Not included	Not included	Not included	Micro-costing	Admissions averted	0
Dan [37]	CEA	Singapore	SARS, H1N1, 1918 Spanish influenza	PPE	Protective measures aimed at only infected patients was the most cost-effective intervention at 23,300 USD per death averted	Dynamic	Multivariate	Healthcare	NR	T, UNDEF	Not included	Not included	Not included	not described	Deaths averted	NR
Halder [38]	CEA	Australia	H1N1	school closure, antiviral	Limited school closure in combination with antiviral treatment was the most cost-effective with 632-777 USD per case averted	Dynamic	Univariate	Societal	NR	T,ADM	Not included	AB	FNM	Micro-costing	Attack rate reduction, cases averted	3
Jamotte [39]	CEA^a^	Australia	H1N1	Vaccination	Quadrivalent, compared trivalent, vaccines were cost-saving and averted almost 70,000 cases per year	Static	univariate	Societal & healthcare	NR	T,ADM	Not included	AB, TR,T	Not included	Micro-costing	Cases averted	NR
Kelso [40]	CEA^a^	Australia	H5N1	school closure, antiviral, workforce reduction, social distancing	A combination of antiviral treatment and prophylaxis, extended school closure, social distancing was most effective and was cost-saving compared to no intervention	Dynamic	Univariate	Societal	Lifetime	T	Not included	AB	Not included	Micro-costing	Attack rates	3
Li [41]	CEA	China	H1N1	Quarantine	Mandatory quarantine in the H1N1 epidemic in China had a cost of 22 USD per case averted which was not considered to be cost-effective^b^	Dynamic	–	NR	NR	T	Not included	ADM	Not included	Not described	Cases averted	NR
Nishiura [42]	CEA	Japan	H1N1	School closure	School closure was not found to be cost-effective with an ICER ranging from approximately 1.5E + 07 to 1E + 11 Yen per Life Year	Dynamic	Univariate	Societal	NR	Not included	Not included	AB	Not included	Micro-costing	Years of life saved	NR
Pershad [29]	CEA	US	H1N1	Screening	Pre-screening in tents compared to no use of tents resulted in 637 USD per percentage point decrease in hospital elopement rate	Trial data	Univariate	Healthcare	NR	T,ADM	Not included	Not included	Not included	Micro-costing	Health care quality indicators	NR
Tsuzuki [30]	CEA	Japan	H1N1	Vaccination	Quadrivalent, compared trivalent, vaccines were cost-saving and averted 528 cases per 100,000	Dynamic	Probabilistic	Societal & healthcare	NR	T,ADM	Not included	AB	FNM	Micro-costing	Years of life saved	2
Wong [61]	CEA	Hong Kong	H1N1	School closure	Individual school closure at the lowest case threshold was the most cost-effective with 1145 USD per case averted	Dynamic	Probabilistic	NR	NR	T	Not included	AB	Not included	Micro-costing	Attack rates	NR
Yoo [32]	CEA	US	H1N1	Vaccination	School located season influenza vaccination resulted in a 12% higher vaccination rate with 36 USD per vaccination	Trial data	Probabilistic	Societal	NR	T,ADM	Not included	AB	Not included	Micro-costing	Proportion vaccinated	NR
Mota [34]	CEA	Brazil	H1N1	Sick leave policies among health care workers	2-day sick leave with reassessment proved to be cheaper and more effective than a 7-day sick leave policy with 609 USD per healthcare worker on leave	Trial data	–	NR	NR	T,AB	Not included	Not included	Not included	Mixed	Days of sick leave averted per 100 health care workers	NR
Gupta [33]	CEA^a^	Canada	SARS	Quarantine	Compared to care as usual and isolation of infected patients, quarantine of infected patients and contacts was cost-saving and reduced transmission	Static	–	NR	NR	T,ADM	Not included	AB	FNM	Mixed	Cases averted	NR
Araz [43]	CUA	US	H1N1	School closure	In the H1N1 scenario, school closure had an ICER between 56,100 to 334,800 USD per QALY gained depending on closure length and transmission intensity	Dynamic	Univariate	Societal	NR	Not included	Not included	AB	Not included	Micro-costing	QALY	3
Beigi [44]	CUA	US	H1N1	Vaccination	Single-dose vaccination in high prevalence scenarios dominated the no vaccination option with decreasing cost-effectiveness with lower prevalence and increased doses	Static	Probabilistic	Societal & healthcare	NR	T	Not included	AB	Not included	Micro-costing	QALY	3
Giglio [48]	CUA	Argentina	H1N1	Vaccination	Vaccination of 6-month old to 5-year old was the most cost-effective with 717 USD per QALY gained	Static	Univariate	NR	NR	T,ADM	Not included	Not included	Not included	Micro-costing	QALY	3
Hibbert [49]	CUA	US	H1N1	Vaccination	Vaccination of children dominated the no vaccination strategy^b^	Trial data	Univariate	Societal	1-year	T,ADM	Not included	AB, PR	Not included	Micro-costing	QALY	0
Khazeni [51]	CUA	US	H7N9, H5N1	Vaccination	Vaccination at 4 months compared to 6 months was cost-effective with 10,689 USD per QALY gained	Dynamic	Univariate	Societal	Lifetime	T	FNRM	AB	Not included	Micro-costing	QALY	3
Khazeni [52]	CUA	US	H5N1	Non defined non-pharmaceutical interventions, Vaccination, Antiviral,	Non-pharmaceutical interventions, vaccination and antivirals in quantities similar to current US stockpiles resulted in 8907 USD per QALY gained compared to no intervention	Dynamic	Univariate	Societal	Lifetime	T,ADM	Not included	AB	Not included	Micro-costing	QALY	3
Khazeni [50]	CUA	US	H1N1	Vaccination	Vaccination in the US population against the H1N1 pandemic in October instead of November would be cost-saving and an additional gain of 9200 QALYs	Dynamic	Univariate	Societal	Lifetime	T,ADM	Not included	AB	Not included	Micro-costing	QALY	3
Lee [53]	CUA	US	H1N1	Antivirals	Initialization of antiviral treatment after PCR confirmed test was the most cost-effective with a difference of 67 USD per QALY to the second most cost-effective strategy and increasing with cost of antivirals	Static	Probabilistic	Societal and healthcare	NR	T,ADM	Not included	AB	Not included	Micro-costing	QALY	3
McGarry [54]	CUA	US	H1N1	Vaccination	PCV13 vaccination compared to PCV7 vaccination was cost-saving and would have prevented 3700 deaths in an H1N1 scenario	Static/mathematical	Univariate	Healthcare	Lifetime	T	FRM	Not included	Not included	Mixed	QALY	3
Sander [55]	CUA	Canada	H1N1	Vaccination	The vaccination program against the H1N1 in Ontario was cost-effective with an ICER of 9140 per QALY gained	Dynamic	Probabilistic	Healthcare	Lifetime	T,ADM	Not included	Not included	Not included	Micro-costing	QALY	5
Xue [45]	CUA	Norway	H1N1	School closure	When simulating a pandemic similar to H1N1 school closure as single intervention would not have been cost-effective with an ICER ranging from 136,427 to 2 192,323 USD per QALY	Dynamic	Univariate	Societal	NR	T	Not included	AB, ES,TR	Not included	Micro-costing	QALY	4
You [46]	CUA	Hong Kong	H1N1	Antivirals	Initialization of antiviral treatment based on empirical assessment alone dominated PCR-guided treatment and a combination of both	Static	Probabilistic	Healthcare	NR	T,ADM	Not included	Not included	Not included	Micro-costing	QALY	3
Prosser [47]	CUA	US	H1N1	Vaccination	Vaccination prior to the H1N1 outbreak was found cost-saving for high-risk groups. For non-risk groups, the ICER varied from 5000-18,000 USD per QALY	Static	Univariate	Societal	1-year	T,ADM	Not included	Not included	Not included	Micro-costing	QALY	3

Treatment costs may include the cost of vaccination if applicable, Absenteeism may include the estimated opportunity loss for students not attending school during school closures and the opportunity cost lost from educational professionals during school closure

Cost abbreviations: *CBA* Cost–Benefit Analysis, *CEA* Cost-Effectiveness Analysis, *CUA* Cost-Utility Analysis, *T* treatment, *A* administrative, *EQ* equipment, *AB* absenteeism, *PR* presenteeism, *TR* travel expenses, *CP* co-payments, *ES* energy savings, *FRM* future related medical costs, *FUM* future unrelated medical costs, *FNM* future nonmedical costs, *NR* not reported

^a^Type of study determined by author as this was not explicitly mentioned in the study

^b^ICERs not presented in article but calculated by author

A dynamic model was used in 19 studies, while 11 studies used a static model. Four studies, all evaluating interventions against H1N1, did not use a transmission model and instead used trial data. One study evaluated the impact of individuals taking own initiative to have less contact with others, thereby aiming to reduce the risk of contracting the disease, in a sensitivity analysis [51].

Of all included studies, 30 conducted at least some sort of sensitivity analysis by varying parameter values. A univariate analysis was conducted in 19 studies, a probabilistic in 10 studies and a multivariate sensitivity analysis in one study [37]. For dynamic models, in which probabilistic sensitivity analysis is inherently difficult due to the parameters in the model being highly inter-dependent, univariate sensitivity analyses on key or all parameters were performed. Only 11 out of the 34 included studies discounted both costs and health benefits.

Nine studies did not mention the perspective used; however, several of those studies did include costs outside the healthcare perspective suggesting the use of a societal perspective. Fourteen studies used a societal perspective and six studies a healthcare perspective. Four studies assessed the costs and benefits from both a healthcare perspective and the societal perspective. One study used a patient perspective [27]. Of the studies stating a lifetime horizon, two included some types of future costs [51, 54].

Among the cost-effectiveness studies the outcome measure varied greatly: five used cases averted as outcome measure, four estimated the reduced attack rates, and two assessed life years lost [30, 42]. The remaining studies all used different outcome measures, including deaths averted [37], averted admissions [36], care quality indicators (such as turn-around time and emergency department recidivism) [29], proportion vaccinated [32], or days of sick leave per 100 healthcare workers [34].

All but two studies included treatment costs within the healthcare sector. Both of the studies that did not include these costs assessed the cost-effectiveness of school closures [42, 43]. Other included healthcare costs were administration costs (19 studies), equipment (two studies) [36, 56], co-payments (one study) [28], and costs due to days of sick leave of healthcare workers (one study) [34]. One study mentioned healthcare costs but subsequently did not define the costs explicitly [37]. Only one study included future non-related healthcare costs [51]. With respect to costs outside the healthcare sector, 24 studies included productivity losses due to short-term absenteeism, transportation (two studies) [39, 45], administration (one study) [41], treatment (one study) [39], presenteeism (one study) [49],and energy savings (one study) [45].

Ten studies included some form of future costs. Eight of these included future productivity losses, one included non-related medical costs [51] and one included related medical costs [54]. No study included more than one type of future costs. The studies that included productivity losses all used the human capital approach, basing calculations on wages and remaining life expectancy. One study included future related medical costs in the form of lifetime disability caused by the illness [54]. Another study included future non-related medical consumption by age based on insurance data in the US [51]. Four of the ten studies including future costs did not discount these costs.

When possible, we assessed the most likely costing method used, based on the (sometimes limited) information provided in the manuscripts. We refrained from labeling the costing method in two studies as the data used for costing were not described. The most common method found was micro-costing, which was used in 27 of the studies. Mixed costing methods using both micro- and gross-costing were the second most frequently used, while gross-costing was third. None of the studies took into account macro-economic effects of a pandemic.

## Discussion

This study identified a substantial number of studies evaluating intervention strategies for important recent major outbreaks in terms of costs and benefits. We found a strong focus on the H1N1 outbreak and a clear bias toward high-income settings. We also found a discrepancy between pharmaceutical and non-pharmaceutical interventions being evaluated. The majority of the studies adopted a societal perspective but its operationalization varied substantially between studies, also in terms of which costs were included in the evaluation. Furthermore, although many studies modeled future health gains, the inclusion of future costs was limited. Also, none of the included studies included non-marginal effects that outbreaks might have on the healthcare sector and the wider economy.

In this study, we presented an overview of economic evaluations in multiple settings without restrictions to certain interventions. This allowed us to create an overview of the methods used in these economic evaluations of strategies to prevent or mitigate the consequences of major outbreaks. Our focus was on the economic aspects, rendering a comprehensive appraisal of the disease and transmission models used beyond the scope of this study. Still, we emphasize the need for high-quality transmission models in producing reliable economic estimations. In our search of the literature we did not find any studies that took into account more disruptive non-marginal effects of pandemics on the healthcare sector and the wider economy. This suggests that there is a gap between the research on the ex-post evaluation of a pandemic taking a macro-economic perspective and ex-post economic evaluations that estimate the impact of specific interventions.

Some limitations of our study need mentioning. First, our search strategy was broad, but may have missed specific studies. It seems unlikely this would have changed our results. Indeed, we believe that the included studies are relevant and form a sample large enough to base our conclusions on. Second, we searched for economic evaluations in relation to specific outbreaks. In particular, the sample of studies included in this review represents outbreaks that were identified as being potentially large threats. Other criteria could have been used for selecting outbreaks and interventions, which would have resulted in a different sample of studies. We cannot generalize to economic evaluations of interventions targeted at other outbreaks. For example, outbreaks, that may have or have had an even larger impact on health and society than the ones included here, may have been evaluated more extensively, potentially leading to different conclusions. Third, included articles were primarily screened by one researcher (KK). Having a second reviewer for all studies would have been more appropriate. Fourth, we encountered some difficulties in extracting the methods used and assumptions made in some studies. Given the level of information provided in those studies, we cannot rule out that some studies or methods were misclassified in this review. A more detailed presentation of the included elements, methods used and the data sources would facilitate the interpretation of the results and add to the transparency as well as the ability to replicate and compare studies.

To the best of our knowledge, there are no previous studies with a similar scope as ours. Previous reviews often applied a narrower scope by either restricting the search for a specific disease or to a specific setting. Pérez Velasco et al. [62] reviewed the strategies against influenza pandemics. Consistent with our results they found an overrepresentation of pharmaceutical interventions in high-income countries. Pérez Velasco et al. also assessed the quality of the included articles in their study, but focused less on variation in methods. A systematic review by Drake et al. [63], focusing on dynamic transmission economic evaluations of infectious disease interventions in low- and middle-income countries, highlighted the lack of reporting parameter values. This was also the case in our review. Drake et al. emphasized the lack in highlighting the uncertainty surrounding cost estimates in modeling studies. In our sample, we found a vast majority of studies using secondary cost data, with a large number of the studies performing a sensitivity analysis of the cost data. Specifically, many studies addressed uncertainty regarding parameters influencing prices or volumes either using uncertainty applied as a proportion of the mean price estimate or uncertainty regarding the mean cost estimates directly obtained. The number of parameters varied in the sensitivity analyses ranged substantially, from all too just a few. A possible explanation for this difference with the findings from the study by Drake et al. is that in our sample the studies mostly originated from high-income settings where the availability of data might be better. Drake et al. [63] proposed a value of information (VOI) framework to address the indicated shortcomings. This was also suggested by Pérez Velasco et al. [62]. VOI analysis may provide insights about potential beneficial areas to conduct further investigation. In addition, other topics could be addressed such as capacity constraints of the healthcare providers, especially in extra resource constrained or vulnerable settings [64]. A major outbreak with a large number of cases will require large efforts in any setting, which may affect the provision of other healthcare services when resources are diverted.

Our results show that there are large differences in the methods used to estimate the costs and benefits of different interventions. These differences can only very partially be explained by differences in the perspective adopted in the studies, as we found large differences within perspectives as well. Therefore, we conclude that there is a need to standardize which costs to include in economic evaluations in this context. Differences in the inclusion of costs will lead to difficulties comparing studies and their results. Moreover, excluding certain cost categories might create biases in results of economic evaluations and can be done strategically. By ignoring real costs, one also risks unwanted or unexpected effects when the intervention is actually implemented.

Another recommendation is to adopt a lifetime time horizon and to include all relevant benefits and costs during that period. This also implies that future costs need to be included in the evaluation. If life is prolonged due to an intervention, the life years gained can not only result in additional contributions to society (e.g., productivity) but may also result in additional costs, such as healthcare consumption and other consumption. Using long time horizons also increases the importance of discounting, which was not performed in all studies including costs beyond the outbreak duration. Not discounting future costs and effects may lead to biases in the results of an economic evaluation and its influence may be profound [65]. As no global standards exist on which costs to include and which rates to use for discounting costs and effects and whether these should be identical presentation of results with and without discounting (at varying rates) and with and without future costs would be a practical approach [66, 67].

The lack of evaluations from non-high-income countries and regions creates difficulties in generalizing the results to other countries and regions. The importance of this issue is emphasized by the fact that most of the burden of communicable diseases still occurs in low- and middle-income settings. The current bias may therefore leave exactly those policy makers who stand to gain most from better evidence on these matters without it.

Previous studies have addressed the challenge of incorporating behavioral aspects into infectious disease models [13, 68]. In the studies we selected, only one performed a sensitivity analysis in which the effect of individuals limiting their contact with others on their own initiative was explored [51]. This is a topic on which further research is needed, including aimed at standardization of how to include such behavioral changes in economic evaluations. Another topic which needs further research is the impact of outbreaks on the broader economy: the so-called disruptive effects. None of the included studies attempted to incorporate these effects, while they may have a substantial effect on the estimated cost-effectiveness of interventions. For instance, Prager et al. [69] estimated the economic costs of a pandemic influenza to amount to a possible $25 billion in the US. When incorporating avoidance and resilience behavior the potential loss grew to $43 billion. Further research is needed to link the outcomes of such studies to economic evaluations focusing on specific interventions. Based on our findings, we suggest that studies should strive toward more comprehensiveness in what they include and more standardization in terms of how to include relevant costs and (health) benefits. Future costs and productivity costs are two areas in which standardization is clearly required. We also emphasize the need for a presentation of all elements of costs and health effects in future studies in a manner that allows readers to scrutinize the data and methods used, and facilitates transferability of results. Adopting reporting standards such as Consolidated Health Economic Evaluation Reporting Standards (CHEERS) statement would be an improvement in this regard [70].

## Conclusions

We note that inclusion of particular costs and benefits may have distributional consequences, also in the context of deciding on interventions aimed at the prevention and mitigation of potential outbreaks. For instance, including productivity losses in the evaluation of an intervention may favor interventions saving or targeted at younger, productive individuals, who participate in the paid labor force. Such distributional consequences should receive due attention, but are not solved by simply ignoring real costs like productivity costs. The increased costs of prolonging life also deserve mentioning in this context. These costs entail not only both costs of consuming healthcare in added life year but also the consumption of non-medical goods. It should be noted that these costs currently often are not included in economic evaluations [71].

Overall, this paper concludes that the evidence base regarding the cost-effectiveness of interventions targeted at preventing or mitigating the effects of major outbreaks at this stage is biased toward specific settings and outbreaks and methodologically diverse. Given the importance of the issue, effort should be taken to improve this.

## Electronic supplementary material

Below is the link to the electronic supplementary material.

Supplementary material 1 (DOCX 15 kb)
