# Supplementary material for: Costs and benefits of interventions aimed at major infectious disease threats: lessons from the literature
Source: Eur J Health Econ. 2020 Aug 13;21(9):1329–50. doi: 10.1007/s10198-020-01218-4 (PMC7425274; doi:10.1007/s10198-020-01218-4)
Supplement: Supplementary file 1 — Supplementary material 1 (DOCX 15 kb) [file 10198_2020_1218_MOESM1_ESM.docx]

**Appendix 1. Search strings**

**SCOPUS:**

(TITLE("Middle East respiratory syndrome coronavirus" OR sars OR n5n1 OR h1n1 OR cholera OR mers-cov OR h7n9 OR ebola) OR ABS("Middle East respiratory syndrome coronavirus" OR sars OR h5n1 OR h1n1 OR cholera OR mers-cov OR h7n9 OR ebola)) AND (TITLE(economic OR cost* OR costing) OR ABS(economic OR cost* OR costing)) AND (TITLE(benefits OR effectiveness OR cost-effectiveness OR cost-benefit OR cost-utility) OR ABS(benefits OR effectiveness OR cost-effectiveness OR cost-benefit OR cost-utility)) AND NOT DBCOLL(medl) AND ( EXCLUDE ( DOCTYPE,"re " ) OR EXCLUDE ( DOCTYPE,"ch " ) OR EXCLUDE ( DOCTYPE,"bk " ) OR EXCLUDE ( DOCTYPE,"sh " ) ) AND ( LIMIT-TO ( PUBYEAR,2018 ) OR LIMIT-TO ( PUBYEAR,2017 ) OR LIMIT-TO ( PUBYEAR,2016 ) OR LIMIT-TO ( PUBYEAR,2015 ) OR LIMIT-TO ( PUBYEAR,2014 ) OR LIMIT-TO ( PUBYEAR,2013 ) OR LIMIT-TO ( PUBYEAR,2012 ) OR LIMIT-TO ( PUBYEAR,2011 ) OR LIMIT-TO ( PUBYEAR,2010 ) OR LIMIT-TO ( PUBYEAR,2009 ) OR LIMIT-TO ( PUBYEAR,2008 ) OR LIMIT-TO ( PUBYEAR,2007 ) OR LIMIT-TO ( PUBYEAR,2006 ) OR LIMIT-TO ( PUBYEAR,2005 ) OR LIMIT-TO ( PUBYEAR,2004 ) OR LIMIT-TO ( PUBYEAR,2003 ) ) AND ( LIMIT-TO ( LANGUAGE,"English" ) )

**PUBMED:**

(Middle East respiratory syndrome coronavirus[Title/Abstract] OR SARS[Title/Abstract] OR H5N1[Title/Abstract] OR H1N1[Title/Abstract] OR Cholera[Title/Abstract] OR MERS-CoV[Title/Abstract] OR H7N9[Title/Abstract] OR ebola[Title/Abstract]) AND (((economic[Title/Abstract] OR cost*[Title/Abstract] OR costing[Title/Abstract]) AND (benefits[Title/Abstract] OR effectiveness[Title/Abstract])) OR cost-effectiveness OR cost-benefit OR cost-utility) AND ( "2003/01/01"[PDat] : "3000/12/31"[PDat] NOT Animals[Mesh:noexp])

**Appendix 2 Excluded articles after full-text review**

| Author | Title | Year | Reason for exclusion |
| --- | --- | --- | --- |
| Ssematimba | Estimating the between-farm transmission rates for highly pathogenic avian influenza subtype H5N1 epidemics in Bangladesh between 2007 and 2013. | 2017 | Animal subjects |
| Sun | Assessment of China's H5N1 routine vaccination strategy. | 2017 | Animal subjects |
| Tran | An Alternative Vaccination Approach for The Prevention of Highly Pathogenic Avian Influenza Subtype H5N1 in The Red River Delta, Vietnam -A Geospatial-Based Cost-Effectiveness Analysis. | 2016 | Animal subjects |
| Pitrelli | Introduction of a quadrivalent influenza vaccine in Italy: a budget impact analysis. | 2016 | Budget impact analysis |
| Zhao | [A cost-benefit analysis of the influenza H1N1 vaccination in the primary and junior school in Shanghai]. | 2011 | Chinese |
| Lewnard | Strategies to Prevent Cholera Introduction during International Personnel Deployments: A Computational Modeling Analysis Based on the 2010 Haiti Outbreak. | 2016 | No cost comparison |
| Cauchemez | Estimating the impact of school closure on influenza transmission from Sentinel data. | 2008 | Effectiveness study |
| Ciavarella | School closure policies at municipality level for mitigating influenza spread: a model-based evaluation. | 2016 | Effectiveness study |
| Xia | Identifying the relative priorities of subpopulations for containing infectious disease spread. | 2013 | Effectiveness study |
| Sander | Is a Mass Immunization Program for Pandemic (H1N1) 2009 Good Value for Money? Early Evidence from the Canadian Experience. | 2010 | Final study included |
| Anparasan | Resource deployment and donation allocation for epidemic outbreaks | 2017 | Methodology paper |
| Jeuland | Incorporating Cholera Vaccine Herd Protection into Economic Cost-Benefit and Cost-Effectiveness Models | 2009 | Methodology paper |
| Park | A real option analysis for stochastic disease control and vaccine stockpile policy: An application to H1N1 in Korea | 2016 | Methodology paper |
| Harling | Leveraging contact network structure in the design of cluster randomized trials. | 2016 | Methodology paper |
| Mubayi | A cost-based comparison of quarantine strategies for new emerging diseases. | 2010 | Methodology paper |
| Phelps | Beyond cost-effectiveness: Using systems analysis for infectious disease preparedness. | 2016 | Methodology paper |
| Praditsuwan | The efficacy and effectiveness of influenza vaccination among Thai elderly persons living in the community. | 2005 | No access |
| Cao | Evaluating the impacts of vaccination, antiviral treatment and school closure on H1N1 influenza epidemic | 2014 | No access (conference) |
| Yarmand | Cost-effectiveness analysis of vaccination and self-isolation in case of H1N1 | 2010 | No access (conference) |
| Yarmand | A simulation-based analysis of different control policies for H1N1 | 2010 | No access (conference) |
| Clemens | When, how, and where can oral cholera vaccines be used to interrupt cholera outbreaks? | 2014 | No access (book chapter) |
| Sandhu | An intelligent system for predicting and preventing MERS-CoV infection outbreak | 2015 | No economic evaluation |
| Sandhu | Smart monitoring and controlling of Pandemic Influenza A (H1N1) using Social Network Analysis and cloud computing | 2016 | No economic evaluation |
| Basili | Swine influenza and vaccines: an alternative approach for decision making about pandemic prevention. | 2013 | No economic evaluation |
| Shim | Optimal H1N1 vaccination strategies based on self-interest versus group interest. | 2011 | No economic evaluation |
| Tracht | Mathematical modeling of the effectiveness of facemasks in reducing the spread of novel influenza A (H1N1). | 2010 | No economic evaluation |
| Tuite | Optimal pandemic influenza vaccine allocation strategies for the canadian population. | 2010 | No economic evaluation |
| Wells | Accuracy, Precision, Ease-Of-Use, and Cost of Methods to Test Ebola-Relevant Chlorine Solutions. | 2016 | No economic evaluation |
| Weng | Early detection for cases of enterovirus- and influenza-like illness through a newly established school-based syndromic surveillance system in Taipei, January 2010 ~ August 2011. | 2015 | No economic evaluation |
| Srivastav | Analysis of a simple influenza A (H1N1) model with optimal control | 2016 | No economic model |
| Dorratoltaj | Epidemiological and economic impact of pandemic influenza in Chicago: Priorities for vaccine interventions. | 2017 | Not based on relevant outbreak |
| Fast | Cost-Effective Control of Infectious Disease Outbreaks Accounting for Societal Reaction. | 2015 | Not based on relevant outbreak |
| Franke | Comparison of two control groups for estimation of oral cholera vaccine effectiveness using a case-control study design. | 2017 | Not based on relevant outbreak |
| Yaesoubi | Identifying cost-effective dynamic policies to control epidemics. | 2016 | Not based on relevant outbreak |
| Gamache | Development and Assessment of a Public Health Alert Delivered through a Community Health Information Exchange. | 2010 | Not evaluating interventions against outbreak |
| Gache | The 2009 A(H1N1) influenza pandemic in the French Armed Forces: evaluation of three surveillance systems. | 2012 | Not quantitative |
| Deans | Influenza vaccines provide diminished protection but are cost-saving in older adults. | 2010 | Review |
| Mogasale | Oral Cholera Vaccination Delivery Cost in Low- and Middle-Income Countries: An Analysis Based on Systematic Review. | 2016 | Review |
| Chocontá-Piraquive | [Cost-effectiveness of vaccinating pregnant women against pandemic influenza in Colombia]. | 2012 | Spanish |
| González-Canudas | [Cost-effectiveness in the detection of influenza H1N1: clinical data versus rapid tests]. | 2011 | Spanish |
| Rosello | Infectious disease risk and international tourism demand | 2017 | Not quantifying impact of intervention |
| Wilson | A national estimate of the hospitalisation costs for the influenza (H1N1) pandemic in 2009 | 2012 | Not quantifying impact of intervention |
